# Class III phosphatidylinositol 3-kinase complex I subunit NRBF2/Atg38 - from cell and structural biology to health and disease

**DOI:** 10.1080/15548627.2021.1872240

**Published:** 2021-01-18

**Authors:** Yohei Ohashi

**Affiliations:** Division of Protein and Nucleic Acid Chemistry, MRC Laboratory of Molecular Biology, Cambridge, UK

**Keywords:** atg38, membranes, nrbf2, pik3c3, ptdins3p, vps34

## Abstract

Macroautophagy/autophagy is triggered by various starvation and stress conditions. The phospholipid phosphatidylinositol-3-phosphate (PtdIns3P) is essential for the formation of the autophagosome both in yeast and mammals. The class III phosphatidylinositol 3-kinase, PIK3C3C in humans or Vps34 in yeast, produces PtdIns3P by phosphorylating the 3ʹ-OH position of phosphatidylinositol (PtdIns). In order to synthesize PtdIns3P for the initiation of autophagy, PIK3C3/Vps34 has a heterotetrameric core, the PIK3C3 complex I (hereafter complex I) composed of PIK3C3/Vps34, PIK3R4/Vps15, BECN1/Vps30, and ATG14/Atg14. A fifth component of complex I, NRBF2 in mammals and Atg38 in yeast, was found and has been characterized in the past decade. The field has been expanding from cell and structural biology to mouse model and cohort studies. Here I will summarize the structures and models of complex I binding NRBF2/Atg38, its intracellular roles, and its involvement in health and disease. Along with this expansion of the field, different conclusions have been drawn in several topics. I will clarify what has and has not been agreed, and what is to be clarified in the future.

## Introduction

**Figure 1.**
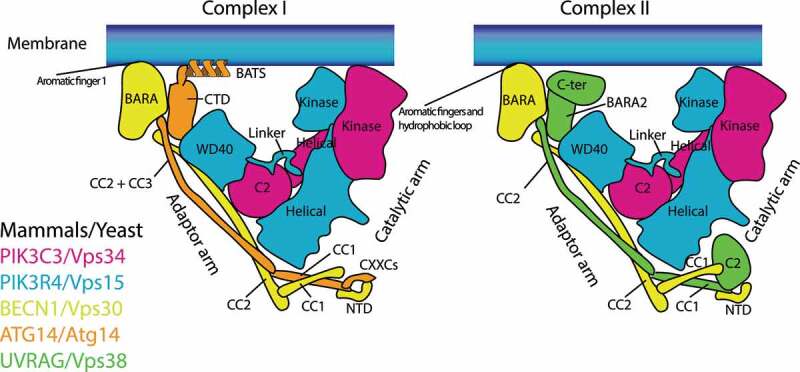
Schematic structural models of mutually exclusive complex I (left) and complex II (right). PIK3C3/Vps34, PIK3R4/Vps15, and BECN1/Vps30 are found in both complexes, whereas ATG14/Atg14 is complex I-specific, and UVRAG/Vps38 is complex II-specific. Complex I associates with membranes via the aromatic finger 1 in the BECN1 BARA domain and the BATS domain in ATG14 (which does not exist in Atg14). Complex II associates with membranes via two aromatic finger motifs (aromatic fingers 1 and 2) and the hydrophobic loop, all of which are in the BECN1 BARA domain. C2: C2 domain; CC: Coiled-coil; BARA: BARA domain; BATS: BATS domain; NTD: N terminal domain; CTD: C terminal domain; CXXCs: CXXC motifs; WD40: WD40 domain

NRBF2 (nuclear receptor binding factor 2) was originally found as an interacting protein of PPAR (peroxisome proliferator activated receptor) [1,2]. Therefore, it was also named comodulator of PPAR and RXRA/RXRα (COPR) 1 and 2 [2]. Later it started drawing attention because of its involvement in autophagy [3]. In 2013, the Ohsumi laboratory isolated the yeast Atg38 as a class III phosphatidylinositol 3-kinase (PtdIns3K, or Vps34) complex I-specific binding protein [4]. Vps34 is a lipid kinase, phosphorylating the 3-OH position of phosphatidylinositol (PtdIns) to generate PtdIns3P [5]. The Vps34 complex I (hereafter complex I) is essential for the initiation of autophagy, and composed of Vps34 (PIK3C3/VPS34 in human), Vps15 (PIK3R4/VPS15/p150 in human), Vps30/Atg6 (BECN1/Beclin1 in human), and Atg14 (ATG14/BARKOR in human), whereas complex II replaces Atg14 with Vps38 (UVRAG in human), having a role on endocytic trafficking (Figure 1). The authors postulated that NRBF2 would be the mammalian Atg38 ortholog. Immediately after this, three groups reported the identification of NRBF2 as a mammalian complex I-binding protein [6–8]. It has been ten years since the NRBF2 involvement in autophagy was found, and the research fields have been expanding from cell biology to structural biology, pathology, and neurobiology.

In this review I will summarize the basic architecture of NRBF2/Atg38, its intracellular roles, and its involvement in health and disease. Naturally, different conclusions among studies also have been seen. In particular, the exact subunit(s) in complex I interacting with NRBF2/Atg38, activation/inhibition of complex I by NRBF2, and the binding stoichiometry between complex I and NRBF2 are topics that have not been agreed upon, and I will discuss what should be clarified.

## Structures of NRBF2/Atg38

**Figure 2.**
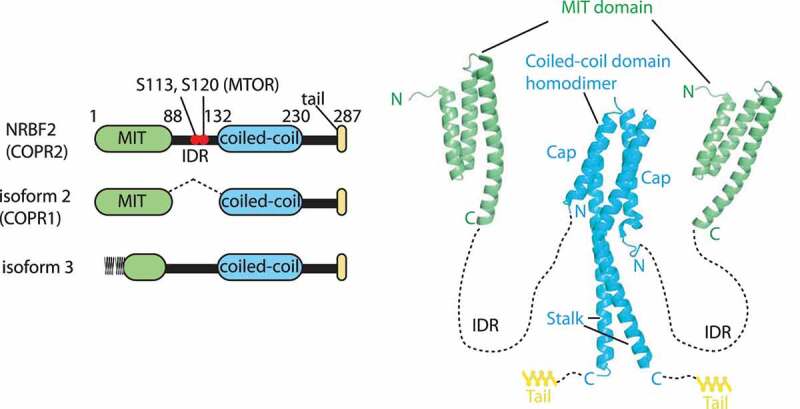
Structures of NRBF2/Atg38. left: schematic representations of NRBF2 and its isoforms (in human residue numbers). The S113 and S120 residues in the intrinsically disordered region (IDR) are phosphorylated by MTOR. Right: structures of the NRBF2 MIT domain (PDB: 4ZEY) and Atg38 coiled-coil domain (PDB: 5KC1). NRBF2/Atg38 makes a stable homodimer via the two segments, the cap and stalk in the coiled-coil domain

NRBF2/COPR2 [2], is composed of a microtubule-interacting and targeting (MIT) domain at its N terminus, and a coiled-coil homodimerization domain at the C terminus. These two domains are flanked by an intrinsically disordered region (IDR, Figure 2 left). There are three NRBF2 isoforms: Isoform 2, also known as COPR1 [2], lacks the IDR, whereas isoform 3 lacks the first two helices of the MIT domain; instead 28 unknown amino acid residues are appended in front of the third helix of the MIT domain (see below). So far there are no reports regarding the physiological roles of these isoforms.

There is good agreement that the MIT domain is responsible for binding to complex I [4,7–10]. The MIT domain is composed of three helices (Figure 2 right). As for isoform 3, although the appended amino acid sequence is not similar to the sequence in the corresponding region of NRBF2, a secondary structure prediction site (HHpred: https://toolkit.tuebingen.mpg.de/tools/hhpred) predicted helices 1 and 2 of the NRBF2/Atg38 MIIT domains to be most similar to the appended sequence, indicating that the N-terminal region of isoform-3 may also have an MIT domain assembly. It would be interesting to see if isoform-3 still binds to complex I, or some other protein(s). Both NRBF2 and Atg38 form strong homodimers via the C-terminal coiled-coil domain [4,8–10] (Figure 2 right). Homodimerization is essential for efficient binding to, and for the integrity of, the complex in yeast and mammals [4,7,10]. The structure of this region is mushroom-like shaped, with a four-helix bundle segment (cap) at its N terminus, and a coiled-coil segment at its C terminus (stalk). Following the stalk, there is a helical extension (tail) both in yeast and mammals. Homodimerization and localization to autophagic puncta of Atg38 were affected when more than two segments of the cap, stalk, and tail were deleted simultaneously [10].

The S113 and S120 residues in the IDR of human NRBF2 are phosphorylated by MTOR (mechanistic target of rapamycin kinase) [11] (Figure 2 left). For the role of these phosphorylations, see the below sections. Although the exact amino acid residue is unknown, NRBF2 is also phosphorylated by ULK1 [11].

## NRBF2/Atg38-binding subunit (s) in complex I – from a methodological point of view

As summarized in Table 1, all of the complex I subunits have been confusingly proposed as NRBF2/Atg38-binding partners. This might be due to the methods being employed, which are mostly immunoprecipitation (IP), hydrogen deuterium exchange mass spectrometry (HDX-MS), or cryo-electron microscopy (cryo-EM). For all methods it is important to bear in mind that except for PIK3C3 in humans [12] and Vps30 in yeast [13], each of the complex I and II subunits is not stable by itself, therefore singly expressed/purified proteins might not be correctly folded, resulting in soluble aggregation. Also, domain deletion can cause conformational changes of the altered protein, as well as the whole complex assembly. IP is the most popular method in cell biology to detect protein-protein interactions. However, because complex I is a stable complex, cellular IPs might not be able to differentiate direct interactions from indirect interactions among the complex I subunits. HDX-MS is a mass-spectrometry based, label-free method, and purified intact proteins can be used to map the interaction sites at 5–10 amino acids resolution [14]. Of note, results should be carefully analyzed to differentiate a direct interaction from indirect conformational changes caused by the direct interaction. Also, for performing HDX-MS and analyzing the data, it is recommended to follow the community’s guidelines [14]. With the resolution revolution in Cryo-EM one is able to routinely visualize macromolecules at 3–5 Å resolution [15]. The resolution depends on various factors including the stability, flexibility, and size of the sample. Since complex I is flexible, and undergoes various conformational changes, the resolution has not been dramatically improved until recently [9,16–19]. Furthermore, NRBF2 and Atg38 are small molecules (32 kDa and 26 kDa, respectively), and it has been difficult to definitively assign their locations to the cryo-EM density [17]. In spite of these technical difficulties, ATG14/Atg14 was found as an NRBF2/Atg38-binding subunit in all studies except for Cao *et al*. [6] (Table 1). This is reasonable, given that ATG14/Atg14 is the complex I-specific subunit. The complex I-Atg38 binding is independent of nutrient condition in yeast [4]. Also, mammalian NRBF2 was identified in non-starvation conditions [6–8,11], indicating mammalian complex I-NRBF2 binding also does not depend on nutrient condition. Ma *et al*. found that residues S113 and S120 in the IDR of NRBF2 are phosphorylated by MTOR in nutrient-rich conditions. The phosphorylated NRBF2 binds to PIK3C3-PIK3R4, making complex I inactive, whereas starvation dephosphorylates these residues, allowing NRBF2 to bind to BECN1-ATG14, leading to an active form of the complex [11]. How the phosphorylated NRBF2 and PIK3C3-PIK3R4 are assembled in rich medium condition remains to be elucidated. Interestingly, previous in vitro studies used bacterially purified NRBF2/Atg38, which are post-translational modification (PTM)-free, and identified Vps30-Atg14 or BECN1-ATG14 as NRBF2/Atg38-interacting subunits [9,10]. These results are consistent with the dephosphorylated NRBF2 interaction with BECN1-ATG14 during starvation by Ma *et al*. [11]. This binding subunit question will ultimately be answered by cryo-EM or crystallography at higher resolution, while various factors such as PTMs and the binding stoichiometry between complex I and NRBF2 (see below) need to be taken into account.

**Table 1.** Summary of NRBF2/Atg38-binding candidates

Reference	Binding subunit (s)	Method
Cao *et al*. [6]	PIK3R4 (WD40 domain), but not PIK3C3, BECN1, nor ATG14	IP in HEK293T cells and *in vitro* translation
Araki *et al*. [4]	Vps34 and Atg14	Yeast two-hybrid and *in vitro* affinity isolation
Lu *et al*. [7]	ATG14, UVRAG	*in vitro* GST affinity isolation (ATG14), HA-NRBF2 affinity isolation from TREx-293 cells followed by peptide identification with mass-spectrometry (UVRAG)
Zhong *et al*. [8]	BECN1, ATG14	IP in HepG2 or HeLa cells
Young *et al*. [9]	BECN1 (BH3 in NTD), ATG14 (CC1), PIK3R4 (Helical)	HDX-MS, EM (negative stain)
Ohashi *et al*. [10]	Vps30 (CC1), Atg14 (CC1)	HDX-MS
Ma *et al*. [11]	PIK3C3-PIK3R4 (when NRBF2 S113 and S120 are phosphorylated), BECN1-ATG14 (when dephosphorylated)	IP with WT, non-phosphorylatable or phosphomimetic NRBF2 and each of singly expressed complex I subunits in HEK293 T cells
Young *et al*. [17]	BECN1 (BH3 in NTD), ATG14 (CC1), PIK3R4 (Helical)	HDX-MS, cryo-EM with complex I (MIT fused to BECN1, MBP-MIT+MIT fused to BECN1, or complex I + FL NRBF2)

Outside of complex I, UVRAG was also found to be an NRBF2-binding protein, and coexpression of PIK3C3-PIK3R4-UVRAG-NRBF2 (without BECN1) increased PtdIns3K activity [7] (Table 1). How NRBF2 modulates the assembly and activity of complex II remains to be seen.

## Binding stoichiometry between complex I and NRBF2/Atg38

**Figure 3.**
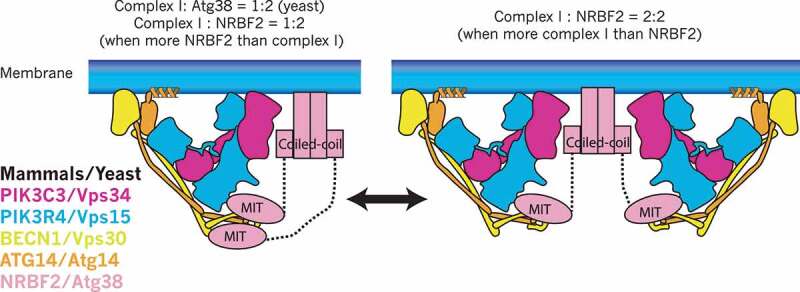
Binding stoichiometry between complex I and NRBF2/Atg38. In mammals, the stoichiometry between complex I and NRBF2 is 1:2 (one copy of complex I and one homodimer of NRBF2, left) when NRBF2 is more abundant than complex I, whereas it can be 2:2 (two copies of complex I and one homodimer of NRBF2, right) when complex I is more abundant than NRBF2. In yeast the stoichiometry is always 1:2 regardless of the comparative abundance between complex I and Atg38. It must be noted that the complex I subunit (S) binding to the MIT domain is still speculative

Both yeast and human complex I can be stably purified without Atg38 and NRBF2 [9,10,19,20], indicating Atg38 and NRBF2 are not essential for the assembly of complex I *in vitro*. On the other hand, both in yeast and mammalian cells, the deletion or knockdown of *ATG38/NRBF2* causes partial dissociation of complex I [4,7,8]. This indicates that there might be a specific step where complex I requires Atg38/NRBF2 for its efficient assembly. The yeast complex I binds to Atg38 with a 1:2 stoichiometry (one copy of complex I bound to one homodimer of Atg38) *in vivo* [4]. Also, for both yeast complex I-Atg38 and human complex I-NRBF2, a study on *in vitro* reconstitution coupled with size exclusion chromatography – multiangle light scattering (SEC-MALS) has been reported [10]. SEC-MALS is a method for determining the absolute molar mass of molecules in solution. This revealed that yeast complex I binds to Atg38 with a 1:2 stoichiometry independent of reconstitution condition (Figure 3). Whereas in the case of the human proteins, the binding stoichiometry depends on the relative abundance of NRBF2 to complex I. When complex I is more abundant than NRBF2, complex I is homodimerized by a NRBF2 homodimer (2:2), whereas the stoichiometry becomes 1:2 when NRBF2 is more abundant than complex I [10] (Figure 3). Structurally, Young *et al*. proposed the 2:2 stoichiometry model and hypothesized that this stoichiometry has important consequences for complex I to interact with membranes [9], whereas the same group also proposed the 1:2 stoichiometry model based on the observation that 2 copies of the MIT domain are required to fully activate complex I [17]. This *in vitro* stoichiometry change remains to be seen *in vivo*. One possibility is that in mammalian cells the binding stoichiometry may be regulated in a spatio-temporal manner depending on the local concentration of complex I and NRBF2.

## Membrane binding role of NRBF2/Atg38

Both yeast and human complexes I and II are Y-shaped, bearing PIK3C3/Vps34 and PIK3R4/Vps15 as the catalytic arm, and BECN1/Vps30 and ATG14/Atg14 (complex I) or UVRAG/Vps38 (complex II) as the adaptor arm [13,16,20] (Figure 1). The BATS domain of ATG14 (which does not exist in yeast Atg14) and the aromatic finger motifs in the BARA domain of BECN1, both of which are in the adaptor arm, are essential for the activity and membrane binding of complexes I and II, respectively [12,13,18,21,22] (Figures 1 and 3). The C-terminal coiled-coil domain of Atg38 is essential for the localization of Atg38 to autophagic puncta and to the pellet fraction in subcellular fractions [10], indicating that this region might be responsible for membrane binding (Figure 3). Since yeast Atg14 lacks the BATS domain, the activity of yeast complex I is much weaker than that of yeast complex II on flat membranes [13], which stands in contrast to human complexes I and II [12]. Therefore, membrane binding may be a more important role for Atg38 in yeast than it is for NRBF2 in mammals. However, the *atg38∆* mutant only reduced, not eliminated, the colocalization of the complex I subunits and Atg18, an effector of complex I to the PAS [4], indicating that there might be unknown factors that help complex I localize to the PAS in addition to Atg38. It is possible that Atg38 might become essential at a specific step during autophagy. It is not clear how NRBF2 contributes to membrane binding in mammals. In rich media, 81% of NRBF2 in HeLa cells is cytosolic (http://mapofthecell.biochem.mpg.de/about.html), suggesting that NRBF2 may become membrane-localized upon starvation.

## NRBF2/Atg38 contribution to PtdIns3P synthesis

It is not clear how Atg38 contributes to PtdIns3P synthesis in yeast. In yeast, PtdIns3P synthesis is solely dependent on Vps34 and Vps15, and deletions of *VPS30, ATG14*, and *VPS38* show negligible effects [23]. Also, the autophagy-defective phenotype of *atg38∆* is milder than that of *atg14∆* [4]. These indicate that Atg38 might not be essential for the production of PtdIns3P, although it is still possible that Atg38 could affect the kinase activity of complex I. On the other hand, mammalian PtdIns3P synthesis is dependent not only on PIK3C3 complexes, but also partially on the class II PI3Ks [24]. Zhong *et al*. measured intracellular PtdIns3P levels, and found that in contrast to yeast, *ATG14* siRNA significantly reduced PtdIns3P levels, whereas *NRBF2* siRNA significantly increased PtdIns3P levels during serum starvation, hypothesizing that NRBF2 is a catalytic suppressor of complex I [8]. The opposite has also been reported by several groups. Lu *et al*. reported that NRBF2 facilitates complex I activity in cells [7]. *In vitro* studies from the Hurley group showed that adding excess amounts of NRBF2 to complex I increased the kinase activity [9,17], although the fold increase in the activation significantly differs between the two papers from this group [9,17] (Table 2). Interestingly, as described above, Ma *et al*. reported that the NRBF2 contribution to the activity depends on the phosphorylation status of NRBF2 by MTOR [11] (Table 2). In contrast to this study, which showed that phosphorylated NRBF2 inactivates complex I by binding to PIK3R4, Young *et al*. reported that bacterially purified, PTM-free NRBF2 activates complex I by binding to the helical solenoid of PIK3R4 [17] (Tables 1 and 2). While the NRBF2 binding to PIK3R4 is a common observation between Ma *et al*. and Young *et al*., how these opposite conclusions were drawn between the cellular (Ma *et al*.) and *in vitro* (Young *et al*.) studies needs to be clarified in the future.

**Table 2.** Summary of NRBF2 contribution to PIK3C3 kinase activity

Reference	activate/inhibit	Method	substrate
Lu *et al*. [7]	activate	IP from mice ±*NRBF2*, or IP with coexpressed PIK3C3-PIK3R4-ATG14 or PIK3C3-PIK3R4-UVRAG ± NRBF2 in HEK293T cells, then detect with TLC	sonicated PI
Zhong *et al*. [8]	inhibit	PtdIns3P detection with ELISA (Echelon) using cell extract from RPE-1 cells (± NRBF2 siRNA)	none
Young *et al*. [9]	activate (10-fold increase for both FL and MIT)	ADP-Glo assay kit (Promega) with purified complex I and NRBF2 (FL and MIT domain) at the ratio of 1:25	sonicated liposomes (∼50–100 nm) containing 22 μM PI
Young *et al*. [17]	activate (2-fold increase for both FL and MIT)	ADP-Glo assay kit (Promega) with purified complex I and NRBF2 (FL and MIT domain) at the ratio of 1:63	sonicated liposomes with PI + PS at 1:6
Ma *et al*. [11]	activate (with non-phosphorelatable), inhibit (with phosphomimetic)	IP with flag-tagged NRBF2 constructs, stably expressed in MEFs, then detect with TLC	sonicated PI (Avanti 840,042 C)
Cai *et al*. [27]	activate	IP with CCZ1 in mouse brains and N2a cells ± NRBF2, then detect with a PtdIns3P ELISA kit (unknown)	Not described

Eukaryotic cells are compartmentalized by biological membranes, mainly composed of glycerophospholipids, phosphatidylcholine (PC), phophatidylethanolamine (PE), phosphatidylserine (PS), and phosphatidylinositol (PtdIns) [25,26]. We recently found that lipid composition and size (membrane curvature) greatly affect the activity of human complexes I and II [12]. None of the above studies has used a lipid substrate with a physiologically relevant lipid composition for the lipid kinase assay (Table 2). How NRBF2 affects complex I activity with a physiologically relevant substrate needs to be seen in the future.

## NRBF2/Atg38 involvement in autophagy

**Figure 4.**
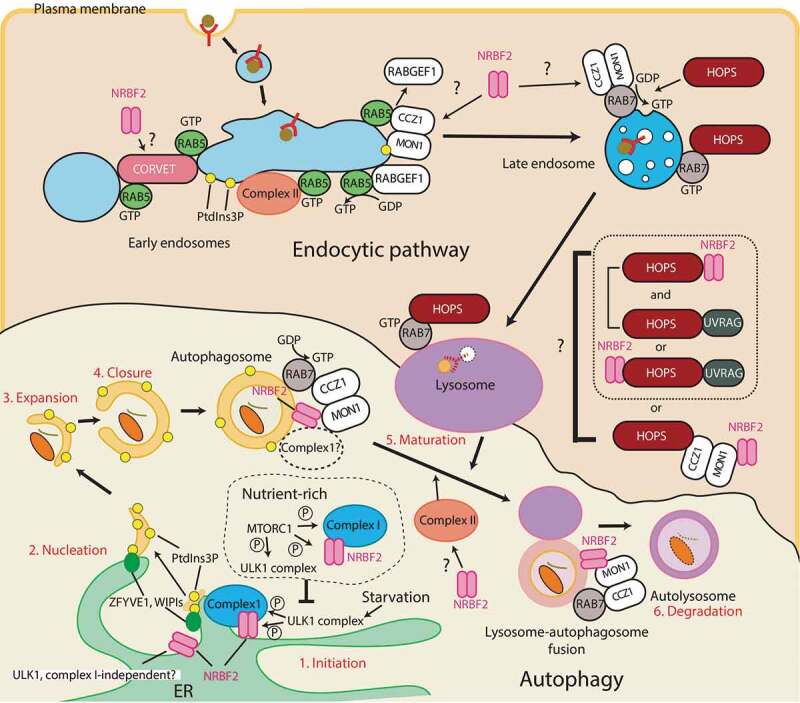
Autophagy and endocytic pathways that involve NRBF2. Autophagy: In nutrient-rich conditions, MTORC1 inhibits autophagy by phosphorylating the ULK1 complex and complex I-NRBF2 complex. Upon starvation, MTORC1 is inactivated, leading to the ULK1 complex activation that recruits complex I-NRBF2, which in turn produces PtdIns3P (1. Inititation). PtdIns3P recruits its effectors such as DFCPs and WIPIs at omegasomes that causes nucleation of phagophores (2. Nucleation). The phagophore expands (3. Expansion), then closes (4. Closure) to become an autophagosome. The autophagosome fuses with lysosomes to become an autolysosome (5. Maturation). The cargos are eventually degraded in the autolysosome (6. Degradation). As well as the initiation step, NRBF2 is also involved in the autophagy maturation step by associating with the MON1-CCZ1 complex. The NRBF2-MON1-CCZ1 facilitates the GEF activity of RAB7, which is involved in the maturation step. Circled P: phosphorylation; ?: the direct involvement of NRBF2 has not been confirmed. Endocytic pathway: During endocytosis, lipids and surface proteins including ligand-receptor complexes are internalized. The endocytosed vesicle first fuses with the early endosome marked by RAB5. The early endosome matures into the late endosome marked by RAB7, then the late endosome eventually fuses with lysosomes to degrade the endocytosed cargos. The early to late endosome transition is mediated by the MON1-CCZ1 complex. The CORVET and HOPS complexes facilitate the tethering of early endosomes and late endosomes, respectively. NRBF2 was found to interact with VPP33A, the common subunit between the CORVET and HOPS complexes. Also, NRBF2 is known to interact with the MON1-CCZ1 complex during autophagy and phagocytosis, but it remains to be seen whether this interaction also occurs in the endocytic pathway or not. The complex II-specific UVRAG subunit is also known to bind to the HOPS complex independently of complex II. It has been unclear whether the NRBF2 and UVRAG interactions with the HOPS complex are mutually exclusive or not

Of the several types of autophagy, here I will discuss macroautophagy (hereafter referred to as autophagy). Autophagy is triggered by starvation, and its process is composed of sequential stages: Initiation, nucleation, expansion, closure, maturation, and degradation (Figure 4, colored in red). The initiation of autophagosome biogenesis is negatively regulated by MTOR complex 1 (MTORC1) that phosphorylates the ULK1 complex [27–30] and complex I [11,31] (also see **NRBF2/Atg38-binding subunit(s) in complex I** and **NRBF2/Atg38 contribution to PtdIns3P synthesis**). Upon starvation MTORC1 is inactivated, which leads to activation of the ULK1 complex. ULK1 complex activation recruits complex I, which produces PtdIns3P at highly curved ER structures called omegasomes (1. Initiation in Figure 4)[32]. The locally enriched PtdIns3P leads to nucleation of phagophores by recruiting downstream effectors such as WIPIs and DFCP (2. Nucleation in Figure 4), which is followed by membrane expansion, closure (3. Expansion and 4. Closure in Figure 4), and autophagosome fusion with lysosomes to become autolysosomes (5. Maturation in Figure 4). Finally, the cargos are degraded (6. Degradation in Figure 4) [33,34]. Complex I and PtdIns3P are essential for the initiation of autophagy both in yeast and mammals [23,35–38]. During starvation or starvation-mimicking conditions, NRBF2/Atg38 colocalizes with autophagosome markers [4,6–8]. NRBF2 knockdown significantly reduced the localization of autophagosome markers and increased the SQSTM1/p62 levels [6,7,11,39]. Also, *atg38∆* reduced the autophagic activity, although it is milder than *atg14∆* [4]. For the above reasons, and assuming NRBF2/Atg38 is a binding protein of complex I that is essentially involved in the initiation of autophagy, it is conceivable that NRBF2/Atg38 is a positive regulator for the initiation of autophagy. On the other hand, Zhong *et al*. reported that NRBF2 is a negative regulator, based on the reduced SQSTM1 levels and increased intracellular PtdIns3P levels in *NRBF2* knockdown cells during starvation [8] (see above and Table 2). Interestingly, the same study also reported that NRBF2 puncta formation can be independent of the ULK1 complex [8], which is essential for the localization of complex I to the autophagosome [40]. This indicates NRBF2 could be involved in the initiation of autophagy either with complex I or in an ULK1 and complex I–independent manner. In *Schizosaccharomyces pombe*, Atg38 is known to interact with Atg8 via an Atg8-family-interacting motif (AIM) which is not conserved in other species. This interaction is important for autophagosome expansion and autophagy flux [41].

Knockout or knockdown of *NRBF2* also impairs the generation of autolysosomes, indicating that NRBF2 can also be involved in the autophagosome maturation step [7,42]. NRBF2 deficiency prohibits RAB7 recruitment to the autophagosome that is thought to be important for the maturation step during autophagy [43]. NRBF2 binds to the MON1-CCZ1 complex, a guanine nucleotide exchange factor (GEF) that activates RAB7 by allowing RAB7 to bind GTP (see the below chapters for more details) [42,44]. The NRBF2-associated MON1-CCZ1 GEF activity is increased in a starvation-induced autophagy condition, and decreased with the MON1-CCZ1 complex purified from *nrbf2*^−/-^ mouse brain [42]. The NRBF2-MON1-CCZ1 interacts with complex I subunits in mouse brains under non-starvation conditions [42]. It remains to be seen at which organelle this interaction occurs, and whether complex I can be also involved in the autophagosome maturation step with NRBF2 during starvation (Figure 4). In contrast to this finding, Kuchitsu *et al*. showed that RAB7 is dispensable for the autophagosome maturation step during starvation [45]. In this case, NRBF2 should have an additional role in the maturation step to show the impairment of the autolysosome generation in the absence of *NRBF2*.

## NRBF2 involvement in the endocytic pathway

The endocytic pathway is essential for keeping cellular homeostasis. This pathway is mainly used for internalizing lipids and surface proteins including ligand-receptor complexes (endocytosis). During endocytosis the internalized vesicle is first fused with the early endosome marked by RAB5, then the early endosome matures into the late endosome marked by RAB7. The late endosome eventually fuses with lysosomes to degrade the content [46] (Figure 4). The transition from early to late endosomes marked by RAB5 and RAB7 is mediated by the SAND-1/MON1-CCZ1 complex (SAND-1 for *C. elegans*, and MON1 for mammals), which is an effector of RAB5 that displaces the RAB5 GEF, RABGEF1/Rabex5, from membranes. At the same time the SAND-1/MON1-CCZ1 complex is a GEF for RAB7 (Figure 4) [47–50]. Membrane fusion on early endosomes and late endosomes/lysosomes are facilitated by the tethering complexes class C core vacuole/endosome tethering (CORVET) and homotypic fusion and vacuole protein sorting (HOPS) complex, respectively [51,52]. These complexes share common subunits (Vps11, Vps16, Vps18, and Vps33), and have unique subunits Vps8 and Vps3 for the CORVET complex, and Vps41 and Vps39 for the HOPS complex [51,52]. On early endosomes, complex II is recruited by RAB5 to synthesize PtdIns3P [53,54]. Also, Vps21 (RAB5 ortholog in yeast) recruits the CORVET complex via Vps8 and Vps3 [55–57]. Whereas the HOPS complex is recruited to late endosomes/lysosomes by RAB7 (Figure 4) [51,52,58]. The HOPS complex also interacts with the SAND-1/MON1-CCZ1 complex [50,51]. SAND1 can bind to PtdIns3P, and the intracellular SAND1 localization is dependent of PtdIns3P [50]. An endocytic pathway BioID study identified the PIK3C3 complex subunits including NRBF2, but not ATG14 as interacting proteins of VPS33A, the core subunit of the human CORVET/HOPS complexes [59]. Because ATG14 was not found in this study, it is likely that this interaction could be independent of complex I. As described above and below, during autophagy and phagocytosis NRBF2 is reported to bind to the MON1-CCZ1 complex, which binds to the HOPS complex. It remains to be elucidated whether BioID detected the direct interaction between NRBF2 and the CORVET/HOPS complex, or the indirect interaction mediated by the MON1-CCZ1 complex (Figure 4). The complex II-specific UVRAG subunit is also known to bind to the HOPS complex independently of complex II [60]. This interaction was found in the maturation steps of autophagy and endocytosis, although its direct involvement in the autophagosome maturation step was not confirmed by Jiang *et al*. [61]. Whether the HOPS complex containing NRBF2 and UVRAG are mutually exclusive or not also remains to be seen (Figure 4).

## NRBF2 in health and disease

According to the Human Protein Atlas (https://www.proteinatlas.org/) and Yasumo *et al*. [1], *NRBF2* mRNA is expressed ubiquitously in human organs, particularly at high levels in the blood, liver, and muscle tissues. The NRBF2 protein can also be detected ubiquitously in all organs (Human Protein Atlas). The Proteomics DB (https://www.proteomicsdb.org/proteomicsdb/#overview) shows that all three NRBF2 isoforms are highly expressed in the placenta, spleen, B-lymphocyte, and natural killer cells. *nrbf2*^−/-^ null mutant mice are viable, and no enhanced mortality, whereas these mice showed focal liver necrosis [7]. Although the phenotype of *atg14* null mutant mice has not been reported, this *nrbf2* KO mice phenotype is very different from the embryonic lethal phenotypes of *pik3c3, pik3r4*, and *becn1* null mutant mice, and the neonatal lethal phenotypes of null mutant mice lacking essential *ATG* genes [62,63]. This indicates that the NRBF2 contribution to autophagic activity during embryogenesis and neonatal stages might be minor compared to other major autophagy players.

### NRBF2 involvement in inflammatory bowel diseases (IBD) and phagocytosis

**Figure 5.**
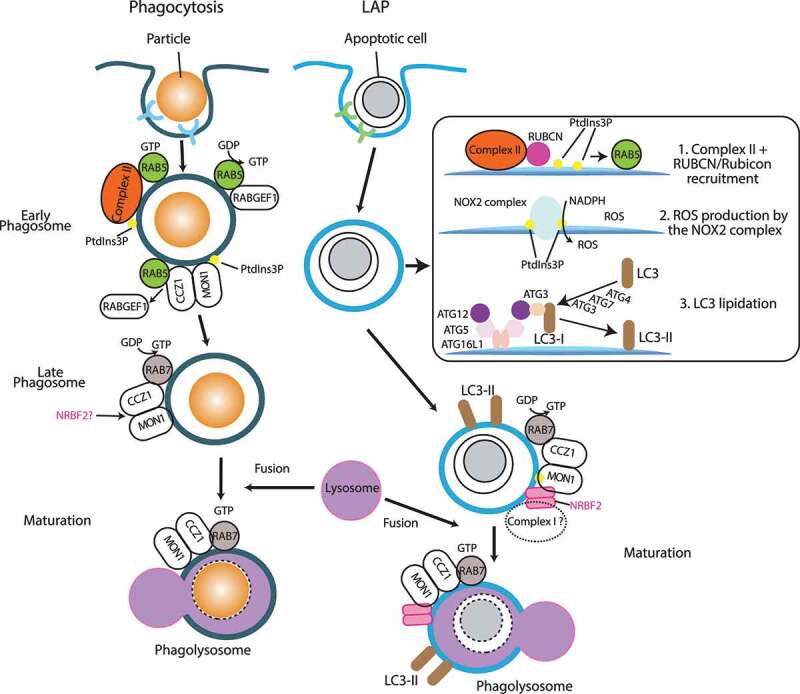
Phagocytosis and LAP pathways where PIK3C3 complexes and NRBF2 are involved. In phagocytosis, the closed phagosome first fuses with early endosomes to become the early phagosome marked by RAB5. The early phagosome matures into the late phagosome, and it eventually fuses with lysosomes to become the phagolysosome (maturation), then the particle is degraded. Similar to the endocytic pathway, RAB5 recruits complex II to the early phagosome, and the RAB5-RAB7 transition is mediated by the MON1-CCZ1 complex. In LAP, complex II and RUBCN/Rubicon are recruited to the closed phagosome (LAPosome, step1), where RUBCN is essential for the recruitment of complex II. In contrast to phagocytosis, the complex II- RUBCN complex is required for the recruitment of RAB5 [74]. The PtdIns3P synthesized by the complex II- RUBCN complex recruits the NOX2 complex, which synthesizes ROS from NADPH on the LAPosome (step2). This is followed by LC3 lipidation (step 3). Along with LC3-II [74], RAB7 activated by the MON1-CCZ1 complex facilitates LAPosome fusion with lysosomes (maturation) to degrade the particle. In BMDM from mice, NRBF2 is required for the maturation step of LAP by facilitating the GEF activity of the MON1-CCZ1 complex to activate RAB7

Maintaining the homeostasis of intestinal epithelial cells is essential for all metazoans, and its failure is one of the causes of IBD represented by Crohn disease and ulcerative colitis [64,65]. Maintenance of the endocytic pathway by PIK3C3 or PtdIns3P is essential for epithelial cell polarity in *Drosophila* and Caco-2 organoids [66], and for polarized distribution of cell-junction proteins in intestinal epithelial cells, because the deficiency of *pik3c3* causes IBD-like features in zebrafish [67]. Inflammation is prevented by removing pathogens, immune complexes, and an excessive number of apoptotic (dying) cells via phagocytosis. Phagocytosis occurs in a series of several steps. First, the particle needs to be recognized by the host cell via specific receptors. Then, the particle is surrounded by the plasma membrane of the host cell, ingested and detached from the plasma membrane. Next, the phagosome fuses with early endosomes followed by late endosomes, transforming from the early phagosome marked by RAB5, to the late phagosome marked by RAB7. The late phagosome eventually fuses with lysosomes, generating a phagolysosome to degrade the particle (Maturation in Phagocytosis, Figure 5) [68,69]. PIK3C3 involvement in the maturation step from early phagosome formation to phagolysosome formation has been shown in RAW macrophages and *C. elegans* [70,71]. Similar to the endocytic pathway, PIK3C3 is recruited to early phagosomes by RAB5. Also, RAB5 recruits the MON1-CCZ1 complex that in turn activates RAB7 (Figure 5; see also **NRBF2 involvement in endocytic pathway**) [47,49,72]. Moreover, the Green laboratory found LC3-associated phagocytosis (LAP), a noncanonical autophagy that can be triggered by various extracellular stimuli such as pathogens, apoptotic cells, immune complex, and viruses [73–75]. LAP requires LC3 and its conjugation (lipidation) system (such as ATG12–ATG5, and ATG16L1, Figure 5), as well as reactive oxygen species (ROS) synthesized by the NOX2 complex (CYBB/NOX2, CYBA/p22^phox^, NCF1/p47^phox^, NCF4/p40^phox^, NCF2/p67^phox^, and RAC1), but the ULK1 complex and ATG9 are dispensable [74]. Also, complex II (but not complex I) along with its specific binding protein RUBCN/Rubicon are essential for the PtdIns3P synthesis on this phagosome (LAPosome) [74]. The recruitment hierarchy of the above protein complexes during LAP is summarized in Figure 5.

Wu *et al*. found that when *nrbf2^−/-^* mice were fed with 2% dextran sulfate sodium (DSS), a chemical colitis inducer where oral administration has been used a model for acute and chronic colitis [76], the mice exhibited an increased inflammatory response, and also a significant accumulation of apoptotic cells in the colon [44]. The phagosomes in bone marrow-derived macrophages (BMDM) from the *nrbf2*^−/-^ mice exhibited increased RAB5A recruitment and delayed RAB7 recruitment, indicating an impairment of the phagosome maturation step. This was due to the reduction in NRBF2 binding to the MON1-CCZ1 complex. The NRBF2-MON1-CCZ1 interaction facilitates the GEF activity of RAB7 (see **NRBF2/Atg38 involvement in autophagy** and **NRBF2 involvement in endocytic pathway**). Also, the authors showed an interaction between the MON1-CCZ1 complex and PIK3C3 [44], although it remains to be seen whether this interaction includes the whole of complex I or PIK3C3 on its own, since PIK3C3 by itself is relatively stable. These phagosomes were stained by LC3, and LC3 staining was increased on the phagosomes from the *nrbf2*^−/-^ mice, indicating NRBF2 regulates the maturation step of LAP [44]. It remains to be seen how LAP, which is driven by complex II + RUBCN, is (potentially) matured by the complex I-NRBF2 complex. Figure 5 summarizes the phagocytosis and LAP pathways where PIK3C3 complexes and NRBF2 are involved.

### NRBF2 in cancer

Autophagy can be either tumor-suppressive or -promoting depending on the stage of tumorigenesis. During the early stage of tumorigenesis, autophagy suppresses tumor-inducing factors such as inflammation, DNA damage, and ROS [77]. The tumor suppressor concept of autophagy was originally derived from the finding that *BECN1* was deleted at high rates in sporadic human breast cancers and ovarian cancers [78]. However, Laddha *et al*. reported that the deletions contain either only *BRCA1*, the well-known tumor suppressor, or both *BRCA1* and *BECN1*, but not *BECN1* alone [79]. This indicates that *BRCA1* deletions are the main drivers of tumorigenesis, and so BECN1’s role as a tumor supressor remains unclear [80]. Indeed, the COSMIC (https://cancer.sanger.ac.uk/cosmic) and ICGC data portal (https://dcc.icgc.org/) databases do not show a significant number of somatic mutations in the *BECN1* locus compared to the loci encoding the other PIK3C3 complex subunits such as *PIK3C3, PIK3R4*, and *UVRAG*. Somatic cancer mutations in the coding regions of PIK3C3 complex subunits, and their potential effects on the stability and activity of the complexes are described elsewhere [81]. During the late stage of tumorigenesis, cancer cells use autophagy to promote their growth and maligancy [77]. Therefore, autophagy has been targeted for cancer therapy.

A survey on the ICGC data portal revealed that *NRBF2* only has five high impact somatic mutations (mutations that change amino acid sequences), which is the lowest frequency among the genes encoding complex I and II subunits, and none of the five mutations are categorized as clinically significant. Mutations in *NRBF2* (in fact all of the PIK3C3 complex subunit-encoding genes) also cannot be found in the Cancer Gene Census (https://cancer.sanger.ac.uk/census) in the COSMIC database. Therefore, NRBF2 is unlikely a cancer driver. Interestingly, the D125N mutation, located at the IDR, has been found from 4 independent samples in COSMIC database [82–84]. The reason why this mutation is enriched remains to be seen. Also, the disease-protective haplotype in one of the correlated, highly trait-associated variants at the 10q21.2 breast cancer risk locus is associated with reduction of the *NRBF2* promoter activity, whereas the other genes encoding PIK3C3 complex subunits were not found in this study [85].

### NRBF2 in neurobiology

Although no somatic mutations associated with neurodegenerative diseases in the coding regions of the PIK3C3 complex subunits have been reported, several studies have implied that the reduced activity and stability of complex I could be associated with neurodegenerative diseases [86–89]. NRBF2 involvement was not mentioned in these studies. The expression levels of *NRBF2*, along with *BECN1, PIK3C3* and *PIK3R4*, are reduced in Alzheimer disease (AD) post mortem brains [90]. A bioinformatics study suggested that gene expression of *RBM8A*, one of the exon junction complex (EJC) subunits, is reduced in AD, which is associated with downregulation of *NRBF2, PIK3R4*, and *BECN1* [91]. For more than two decades, amyloid plaque, composed of amyloid beta (Aβ), has served as a hallmark of AD. NRBF2 is involved in the degradation of Aβ and amyloid beta precursor protein C-terminal fragments (APP-CTFs) in cell models and mice models for AD [39,42,90]. Although this is beyond the scope of this review, the majority of clinical trials targeting Aβ as an AD treatment have failed even when amyloid plaques are eliminated by drug treatment. This indicates that Aβ could be a clinical feature, rather than a cause of the disease [92,93]. In sharp contrast, Aβ has also been shown to have antimicrobial properties [94–96]. Loss of *NRBF2* also causes memory and long-term potentiation deficits in mice [90,97]. It is not clear whether these learning and memory impairments also depend on complex I or not.

### NRBF2 in health

As mentioned above, *NRBF2* mRNA expression is high in the blood and muscles. *NRBF2* mRNA expression level is increased after cycling exercise [98]. A significant number of single nucleotide polymorphisms (SNPs) were found in the *NRBF2* gene of Tibetan pigs, which are adapted to high altitude, compared to Wuzhishan pigs, a low altitude control breed [99]. It has also been reported that hypoxia induces SUMOylation of PIK3C3, which enhances the assembly of complex I [100]. It will be interesting to see whether NRBF2 is actively involved in this assembly or not.

The SNPs in *NRBF2* were also found to be associated with plasma fatty acid metabolism, especially γ-linoleic acid, one of the Omega6 (n6) polyunsaturated fatty acids (PUFAs) [101,102].

## Concluding remarks

In spite of its stable interaction with complex I, *nrbf2* KO phenotypes in cultured cells and mice are less severe than the strong KO phenotypes of the other complex I subunits. This indicates that NRBF2/Atg38 may become important at specific steps in autophagy, or in specific organs in mammals – as indicated by its potential involvement in IBD. Alternatively, NRBF2/Atg38 may become essential in the absence of some other components. As mentioned above, several discrepancies such as the identity of the NRBF2/Atg38-binding subunit(s) in complex I, activation/inhibition of complex I by NRBF2, and the binding stoichiometry are likely to be derived from the methods and experimental conditions that the researchers employed. Also, the NRBF2 effect on the GEF activity of the CCZ1-MON1 complex has been examined only by using immunoprecipitated MON1-CCZ1 complex [42,44]. The GEF assays will be ideally performed with purified recombinant CCZ1-MON1 complex to exclude the possibility of the contribution of other endogenous proteins coimmunoprecipitated with the MON1-CCZ1 complex to the GEF activity. I hope this review will help raise awareness of these issues for researchers so that they might be solved by future research. Additionally, there have been no hereditary diseases reported for which NRBF2 could be responsible. The recent and rapid development of information technology and the development of human disease databases may be able to reveal these.

## Figures and Tables

**Table:** Abbreviations

AD	Alzheimer disease
ATG	autophagy related
ATG14	autophagy related 14
Atg38	autophagy related 38
BECN1	beclin 1
BMDM	bone marrow-derived macrophages
CORVET	class C core vacuole/endosome tethering
Cryo-EM	cryo-electron microscopy
HDX-MS	hydrogen-deuterium exchange mass-spectrometry
HOPS	homotypic fusion and vacuolar protein sorting complex
IBD	inflammatory bowel disease
IP	immunoprecipitation
LAP	LC3-associated phagocytosis
LAPosome	LAP-engaged phagosome
MIT	microtubule-interacting and targeting
MTORC1	mechanistic target of rapamycin kinase complex 1
NRBF2	nuclear receptor binding factor 2
PAS	phagophore assembly site
PIK3C3	class III phosphatidylinositol 3-kinase
PIK3R4	phosphoinositide-3-kinase regulatory subunit 4
PtdIns3K	class III phosphatidylinositol 3-kinase
PTM	post-translational modification
ROS	reactive oxygen species
TLC	thin layer chromatography
ULK1	unc-51 like autophagy activating kinase
UVRAG	UV radiation resistance associated
VPS	vacuolar protein sorting
